# Application of fused deposition modeling (FDM) on bone scaffold manufacturing process: A review

**DOI:** 10.1016/j.heliyon.2022.e11701

**Published:** 2022-11-22

**Authors:** Rochmad Winarso, P.W. Anggoro, Rifky Ismail, J. Jamari, A.P. Bayuseno

**Affiliations:** a Department of Mechanical Engineering, Faculty of Engineering, Universitas Muria Kudus, Indonesia; b Department of Industrial Engineering, Faculty of Industrial Technology University of Atma Jaya Yogyakarta, Yogyakarta, Indonesia; c Department of Mechanical Engineering, Faculty of Engineering, Diponegoro University, Indonesia

**Keywords:** Fused deposition modeling, Porous Scaffold, Bone tissue engineering, 3D printing

## Abstract

Some of the health issues that are becoming more prevalent each year include bone disease and fractures. Because the natural healing process of bones takes a long time, a bone grafting procedure is required so that the patient’s condition can improve rapidly. Because bone grafting procedures such as autographs, allographs, and xenografts have limits, bone replacement is constructed by employing biomaterials in the form of a bone scaffold via additive manufacturing. As a result, fused deposition modeling (FDM) is a proposed technology for the manufacturing process because it is straightforward, capable of producing complex parts and adjustable shapes, and has minimal operational expenses. However, implementing this technique is challenging because of the scarcity of biocompatible and bioactive materials that are suited. This technology has a number of limitations, including a limited variety of biocompatible and bioactive materials, the most appropriate microarchitecture of bone scaffold, and the establishment of printing parameters that can produce bone scaffold with the strongest mechanical properties. This article discusses current advancements in the use of FDM technologies for bone scaffold production.

## 1 Introduction

A bone graft is the world’s second most popular transplanting tissue. More than 500,000 bone grafting procedures are performed in the United States each year, with 2.2 million performed worldwide [1]. Injury, trauma, nonunion after a fracture, infection or anomalies can all result in substantial bone defects, which can lead to long-term malformations such as limb shortening and decreased bone structure and function [2]. Human bones are vulnerable to damage from a variety of sources, including fractures, illnesses, and infections. After trauma and pain, bones have an extraordinary ability to rebuild and heal themselves. Even so, they will not be restored for serious flaws, necessitating external intervention [3]. In particular, bone is separated into two types: spongy cancellous bone and harder cortical bone. It consists of organic components, such as the protein collagen, and inorganic mineral phases, such as hydroxyapatite, which strengthen the entire framework. A human body includes roughly 270 bones, some of which fuse together until about 206 bones remain well into adulthood. Here, the major components of bone are arranged in a hierarchical sequence and range in size from centimeters to nanometers [4].

**Figure 1:**
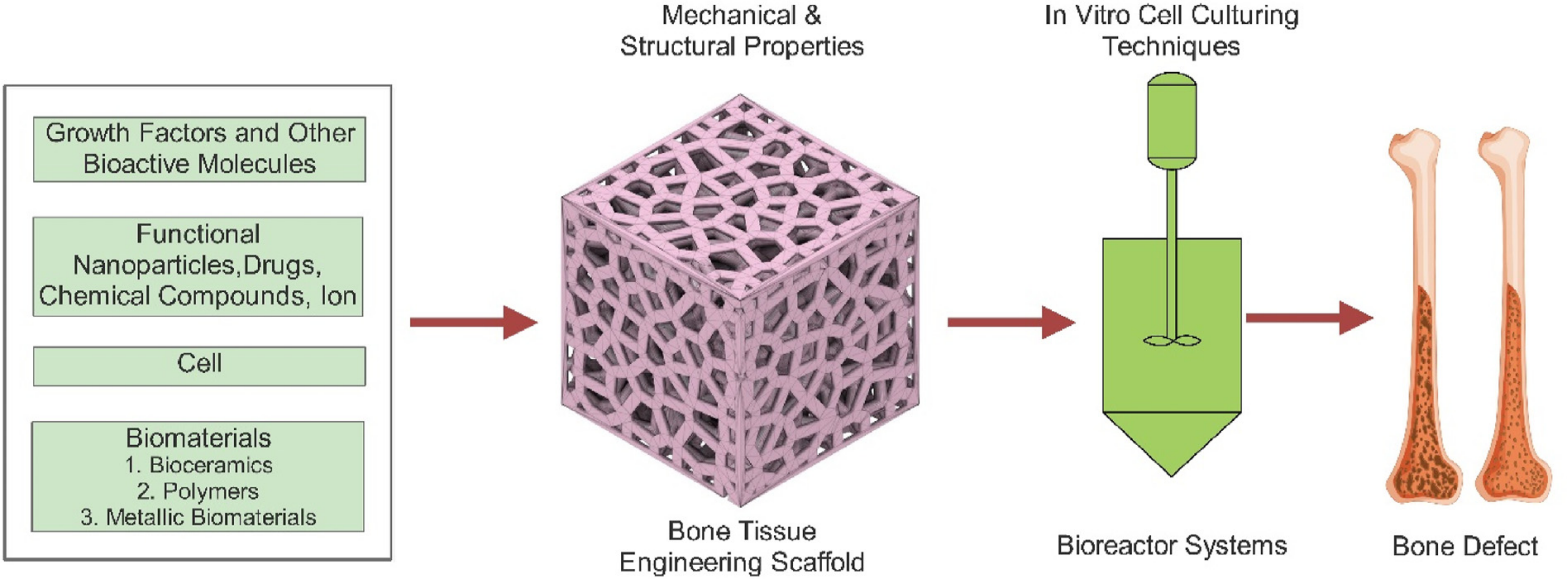
Bone tissue engineering strategy [8].

As a result, bone grafting has emerged as an effective therapeutic strategy for rebuilding and repairing damaged bone tissues in order to overcome bone-destructive causes. Autografting, allografting, and xenografting are the three bone-grafting processes [5, 6]. Autografts obtained from the patient’s own body are considered the gold standard in bone restoration. The autograft, on the other hand, has a relatively small size. Furthermore, removing the autograft results in further surgical trauma and a significant risk of morbidity at the donor site. Allografts derived from other people are a better option than autografts, which are more common. They almost invariably lead to disease transmission and immunological rejection. As a result, innovative bone replacements for surgical bone tissue repair are in high demand [7]. In comparison to more classic bone grafting methods like autografts or allografts, bone tissue engineering (BTE) methodologies (Figure 1) show promise in replacing missing or damaged bone tissue [8].

Tissue engineering, on the other hand, has promised bone regeneration by mixing cells, scaffolds, and biofactors. A bone scaffold is a three-dimensional matrix that allows and promotes osteoinductive cells to attach and grow to its surfaces [9]. Mechanical, biological, and structural properties are the main considering characteristics of a scaffold. Here elastic modulus, compressive strength, and sufficient stiffness are examples of mechanical properties. Biological properties may include biodegradability, bioresorbability, biocompatibility, and non-toxicity. High porosity, pore, interconnectivity, hierarchical structure, and nano topography are structural qualities. Whole-scaffold design (uniform, functional graded, and topological optimization) and cell design units (Voronoi, TPMS), body-centered cubic (BCC), face-centered cubic (FCC), polyhedron, and honeycomb [4] are examples of bone scaffold design variations. The mechanical characteristics of trabecular bone are determined by the porosity and microarchitecture attractiveness of individual trabeculae. The mechanical properties of human bone can be summarized in Table 1 [2].

**Table 1:** Mechanical properties of human bone [2].

Bone properties	Trabecular	Cortical
Porosity (%)	50.00–90.00	1.00–20.00
Young’s modulus E (GPa)	0.05–0.10	17.00–20.00
Compressive strength (MPa)	5.00–10.00	131.00–224.00
Tensile strength (MPa)	1.50–38.00	35.00–283.00
Elongation at break (%)	0.50–3.00	1.07–2.10

Much research has recently been conducted on bone substitute biomaterials, which are used to treat bone abnormalities or fractures. Numerous materials have been studied over the years under the three major material classifications: metals, ceramics, and polymers [4]. As a result, biomaterials are divided into three categories: metal materials (such as titanium and its alloys), inorganic materials (such as bioactive ceramics, hydroxyapatite, and others), and organic materials [10]. Several biomaterials with varying compositions can be used to create scaffolds that mimic the ECM and support the formation of new bone tissue [11]. Kalsi et al. [11] classified biomaterials as natural polymers such as collagen, chitosan, silk fibroin, alginate, hyaluronic acid, and peptide hydrogels, and synthetic polymers such as polyesters and copolymers. Ceramic may also include bioglass, calcium phosphate, and corals. Metal scaffolds, on the other hand, such as Ti-6Al-4 V, Co-based alloys, and stainless steel 316 L, may be used. Swain et al. [12] investigated the effect of sintering temperature on densification and the resulting mechanical, electrical, and biological properties of mechanochemically treated hydroxyapatite (HAp) samples.

Manufacturing processes are further classified into five types: subtractive, additive, joining, splitting, and transformative [13]. Subtraction and additive manufacturing are the two methods for creating bone scaffolds. Subtractive manufacturing refers to any method of producing a bone scaffold by removing a portion of the material from a solid or liquid uniform block. All technologies that develop the porosity geometry of the scaffold by gradually adding matter, frequently layer by layer, without the use of an organic solvent are referred to as additive manufacturing [14].

## 2 Additive manufacture

Additive manufacturing (AM), also known as solid freeform fabrication (SFF) or rapid prototyping (RP), was invented in the late 1980s [6]. AM technologies based on three-dimensional (3D) models are used to build complex structures layer by layer. In contrast to subtractive manufacturing methods, this technology has resulted in complex designs. In non-technical contexts, 3D printing and additive manufacturing (AM) are frequently used interchangeably [15, 16]. AM is a method of material composition that involves fusing, binding, or hardening liquid resins and powders. AM processes are characterized by acronyms such as rapid prototyping (RP), rapid manufacture (RM), 3D printing (3DP), direct digital manufacturing (DDM), and solid freeform fabrication (SFF) [13]. AM is made up of three basic steps: A digital 3D solid model is used to generate an AM file format, such as the traditional standard tessellation language or the newer additive manufacturing file format. The file is then sent to an additive manufacturing machine, where it is adjusted, such as relocated, orientated, or scaled, and the part is built layer by layer [17]. AM can produce parts with complex shapes with minimal post-processing and can work with a wide range of materials, including plastics and metals.

**Figure 2:**
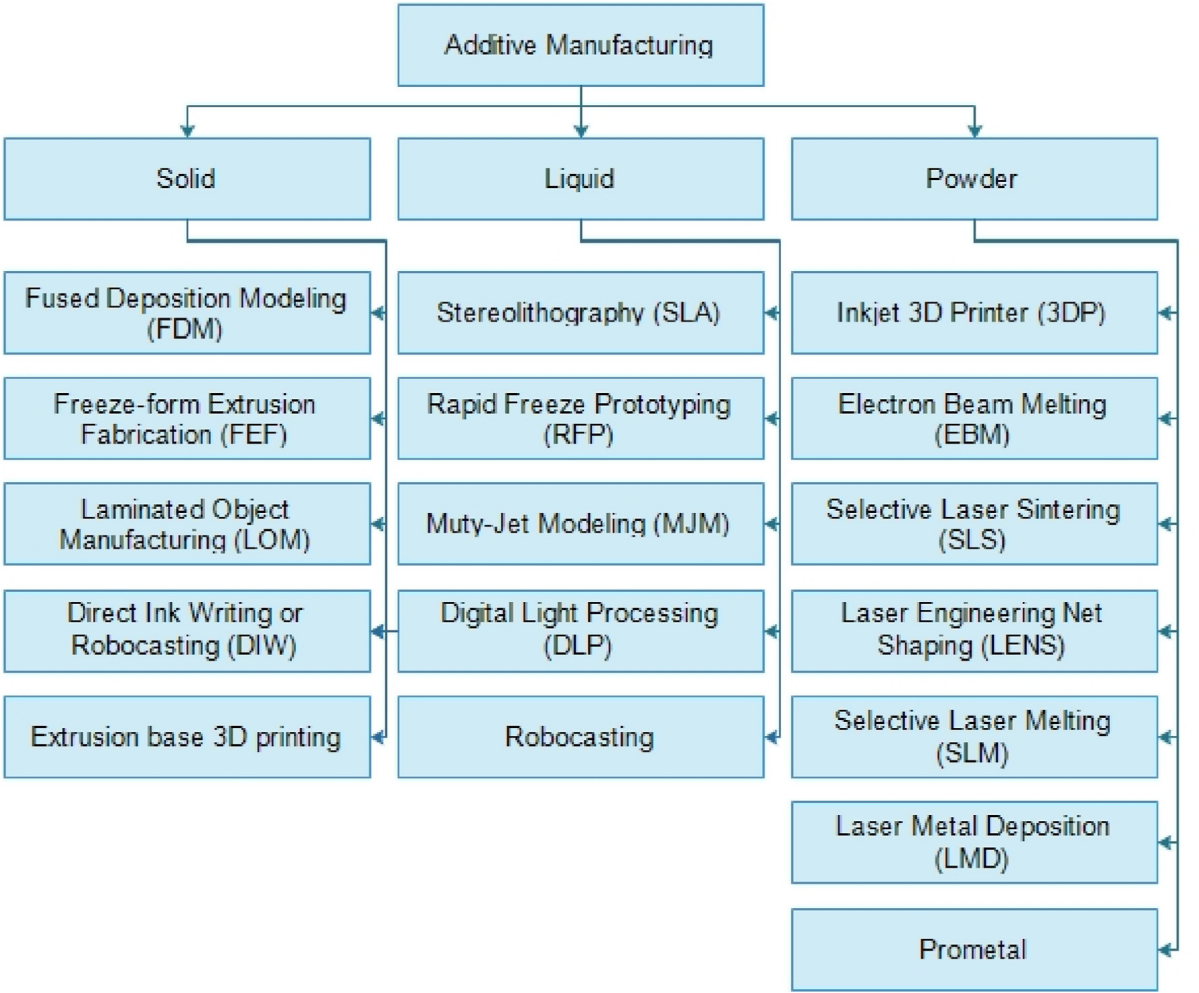
AM process classification based on raw material status [13].

Additive manufacturing has recently increased its market share and extended into new markets such as automotive, medical, and aerospace. This rate of growth is expected to continue in the next years [18]. Based on the material utilized, AM processes can be summarized and categorized. Figure 2 shows a summary of current AM methods based on the material type used [13].

Here, SLA or vat photopolymerization, selective laser sintering (SLS) or powder bed fusion, fused deposition modeling (FDM) or extrusion-based approach, binder jetting (BJ), or 3D powder printing are the most popular and extensively utilized AM techniques today. SLA uses the photopolymerization technology, which includes exposing liquid photopolymer material to UV or laser light selectively. It layers the material via a crosslinking chain reaction [19]. SLS uses a laser to sinter powdered material. The laser heats the material to fusion temperature, resulting in the formation and connection of the object’s layers [20]. Moreover, direct ink writing (DIW) uses a piston, a screw, or pneumatic power to squeeze viscoelastic inks out of the nozzle [21]. Fused deposition modeling (FDM) is a method of building three-dimensional structures by depositing thermoplastic material onto a substrate in layers using a temperature-controlled printhead. A heated printhead melts a thermoplastic filament, allowing for precise successive printing of microscopic layers of semi-molten polymers like polycaprolactone [22]. FDM is the most widely used AM technology for producing functional components because it produces clean and detailed parts in a safe office setting [23]. FDM has several advantages in scaffold fabrication, including safe and efficient operating techniques, good durability, low cost, good accuracy, low energy consumption, low temperature, and the ability to make thermoplastic items with complex geometries [23, 24]. According to the literature, FDM is the only technology used to create customized catheters with promising results [25]. FDM has the potential to be used to make surgical tools, implants, orthoses, and prostheses [26]. Table 2 compares the advantages and disadvantages of various AM methods [13, 23].

**Table 2:** Comparison of various AM methods [13, 23].

Method	Material	Cost	Accuracy	Energy Consumption	Multiple Materials	Temperature
Selective Laser Melting (SLM)	Metal	High	High	High	Fair	High
Electron-Beam Melting (EBM)	Metal	High	High	High	Fair	High
Selective Laser Sintering (SLS)	Polymer, Ceramic, Metal	High	Limited	High	Fair	Low
Fused deposition modeling (FDM)	Polymer	Low	Good	Low	Fair	Low
Robocasting or direct ink writing (DIW)	Polymer, Ceramic, Metal	Low	Good	Low	Good	Low
Stereolithography (SLA)	Resin	High	High	Very Low	Good	Very Low
Laser Engineered Net Shaping (LENS)	Metal	High	Low	High	Fair	High

## 3 Fused deposition modeling

**Figure 3:**
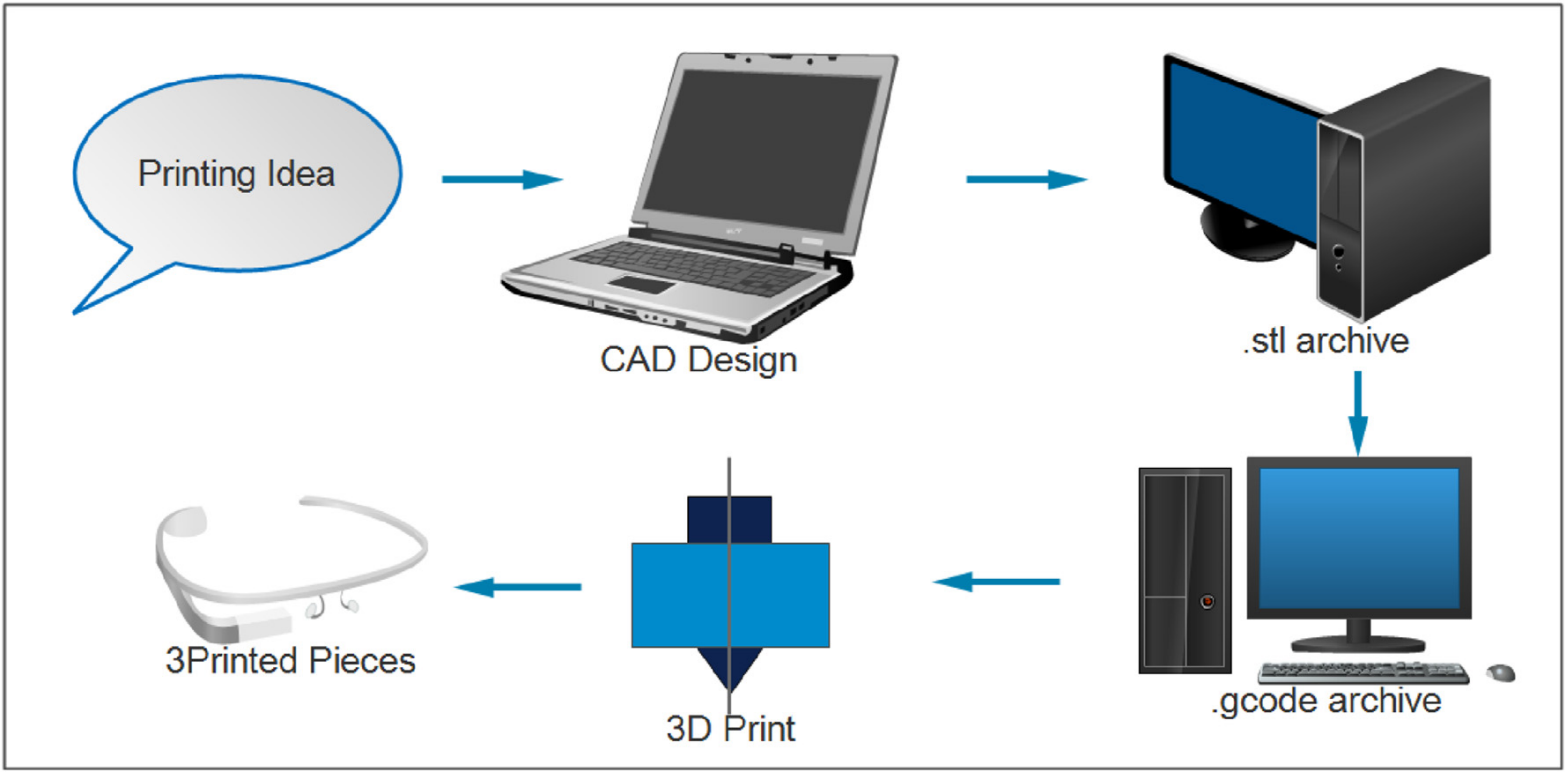
The manufacturing process of FDM [29].

FDM is a 3D printing technology that uses filament extrusion to manufacture 3D components directly from a CAD model [24]. The method was developed and sold in the early 1990s by the Stratasys corporation in the United States. This process layers material on a heated plate by extruding molten material through a specified diameter nozzle. This method has been used to manufacture a number of materials over the years, including polymers, metal powder, ceramics, and composites [27]. Moreover, FDM is one of the most extensively used 3D printing technologies in biomaterial research due to its low cost, small size, ability to produce complex structures, and lack of organic solvents [28]. The first stage in FDM is to use CAD software to create a virtual model of the printed object in the “.stl” archive, then convert the STL archive to a G-code archive. This data is transferred to a printer, which duplicates the design layer by layer until the full model is obtained. The FDM process is summarized in Figure 3 [29].

**Figure 4:**
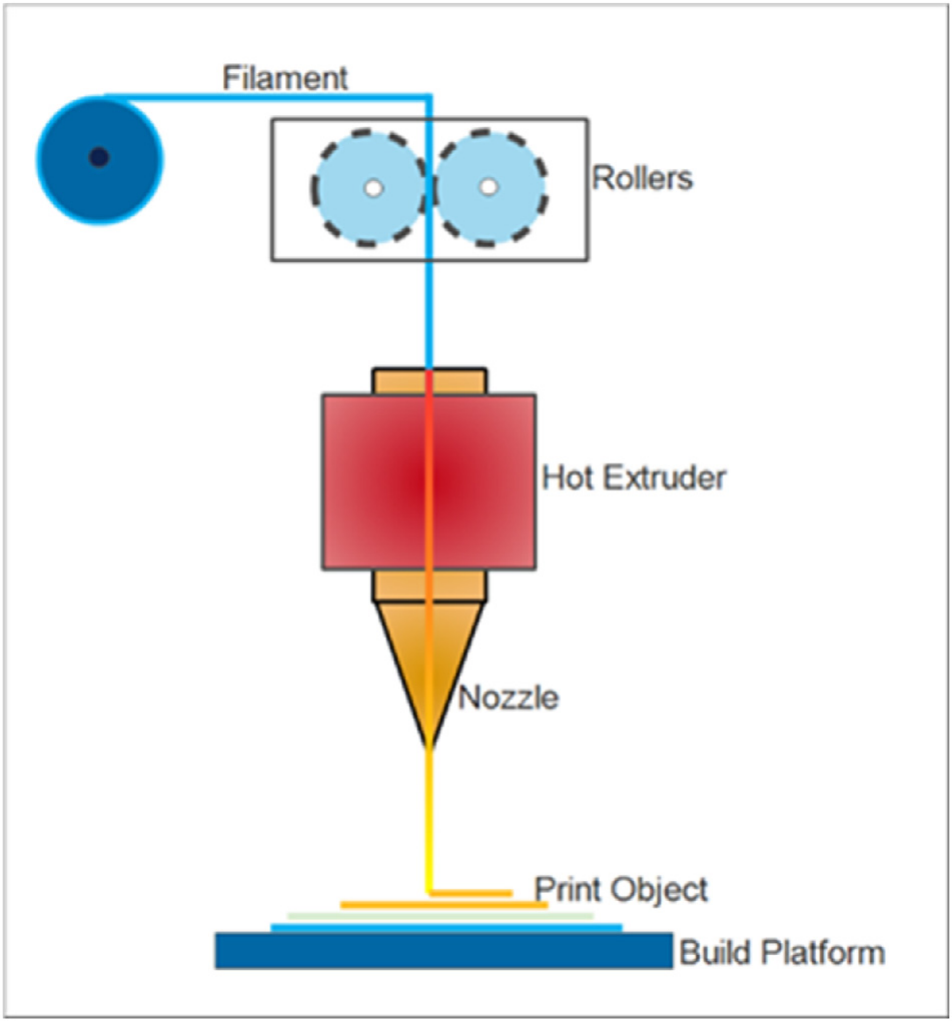
FDM working principle [30].

To create a 2D layer in the head, the heated filament is extruded in molten form onto a platform. Stacking two-dimensional layers yields a three-dimensional part that embodies a design specification [30]. The FDM operational idea is depicted in Figure 4.

Several factors have a significant impact on the quality of the construction part and its manufacturing efficiency. Layer thickness, raster angle, build orientation, infill density, printing speed, infill pattern, extrusion temperature, raster width, nozzle diameter, contour width, contour to contour air gap, contour numbers, air gap, and other factors are all significant [23]. FDM printers use heated polymer filaments released from the nozzle. Because these filament materials are thermoplastic polymers, they may be heated to melt and soften and then chilled to restore their qualities. Furthermore, depending on whether they will be used as a commodity, engineering prototype, or high-performance item, materials used in FDM extrusion may be semi-crystalline or amorphous [31]. Three factors must be considered when using the FDM method to create bone scaffolds: design architecture, materials, and process parameters.

## 4 The design architecture bone scaffold

A transplanted tissue scaffold should degrade quickly. It should also allow cells to produce an extracellular matrix without harming human organs. Degradation is balanced by increasing the strength of the newly formed extracellular matrix, which is the most significant and critical parameter in scaffold design (ECM). Scaffolds made of bone must be robust, malleable, and rigid enough to sustain a wide range of loads. The challenge is to provide mechanical strength adequate for orthopedic applications such as bone and cartilage [32]. The mechanical properties of the bone scaffold decreased considerably as the porosity of the scaffold increased [33]. Additionally, the scaffold must be mechanically strong, with high porosity, specific surface, and pore structure. The fluid permeability of the scaffold is regulated by pore size and interconnectivity [34]. Smaller pore sizes may cause cell obstruction, low permeability, and high strength. Large pore size, on the other hand, may result in poor specific surface area, limited ECM synthesis, low strength, and inadequate cell bridging. As a result, the optimal pore size has been proposed to be between 100 and 600 mm to provide excellent results in a new cell or bone production, permeability, mechanical strength, and vascularization [19]. The design of microarchitecture is a key stage in the creation of bone scaffolds. There are two types of bone scaffold architecture. The first is built on unit cell designs, whereas the second is built on whole designs. Non-parametric designs have some conventional geometry, however, parametric designs are more standardized because they are all developed with specific algorithms. The most commonly used non-parametric patterns for bone scaffolds include BCC, Diamond or FCC, Polyhedron, and Honeycomb. Uniform design, gradient design, and topology optimization (TO) based design are the three types of whole designs [4].

### 4.1 Whole design bone scaffold

In recent years, many research efforts have been dedicated to microarchitecture complete design types and the manufacture of bone scaffolds using FDM. The microarchitecture of the overall design bone scaffold is depicted in Table 3. Serra, Planell, and Navarro [35] study the impact of orthogonal layer configuration (ORTH) and displaced double-layer design (DISPL) on mechanical and biological properties using PLA/PEG/G5 composite materials. The compressive modulus was reduced by modifying the scaffold design from ORTH to DISPL, according to the compression test results. Karuppudaiyan and Singh [36] construct scaffolds with regulated internal architecture and assess their compressive strength and structural modulus using FDM. Four distinct raster laydown patterns have been designed for this project: 0/90°, 0/60/120°, 0/45/90°, and 0/45/90/135°.

**Table 3:** Microarchitecture of whole design bone scaffold.

Author	Design	Material	Porosity (%)	PS (μm)	CS (MPa)	ME (MPa)
Serra, Planell, and Navarro [35]		PLA/PEG				
		ORTH	75 ± 0.86	165 ± 5	NA	92.32 ± 2.18
		DISPL				28.38 ± 3.99
				
		PLA/PEG/G5	70 ± 1.2	165 ± 5	NA	99.81 ± 3.55
		ORTH				44.19 ± 2.67
		DISPL				
Karuppudaiyan and Singh [36]		0/90°	82.17	NA	2.5	57.20
		0/60/120°	82.37		1.76	29.90
		0/45/90°	65.66		9.35	149.75
		0/45/90/135°	62.41		7.35	212.21
Pecci et al. [37]		PLA	27	400		
		layer height 0.25	46	500		
		mm	65	600	27.8 ± 3.5	1.3 ± 0.2
		0.4 mm	35	Random 400–600	25.3 ± 1.5	1.6 ± 0.2
Sohrabian et al. [38]		PLA				
		0/90	60	300	6.289 ± 0.115	230.44 ± 11.2
		0/45/135/90			6.261 ± 0.097	159.19 ± 6.8
		0/60/120			6.253 ± 0.134	202.2 ± 9.5
		0/90 shifted			4.418 ± 0.086	156.56 ± 7.3
		0/45/135/90 shifted			6.151 ± 0.126	177.62 ± 8.3
		0/60/120 shifted			6.427 ± 0.144	179.5 ± 8.1
Bagwan et al. [39]		PLA				
		0/-90/-0/-90	41.32	350	NA	21,871 to 30,948
		0/-30/-0/-30	42.69	350		
		0/-45/-0/-45	42.25	350		
		0/-60/-0/-60	42.70	350		
		0/-45/-90/-135	42.15	350		
		0/-60/-120/-180	42.74	350		
		0/- 90/-0/- 90	47.83	514		20,340 to 28,781
		0/- 30/- 0/- 30	49.14	514		
		0/- 45/- 0/- 45	48.92	514		
		0/-60/-0/-60	49.10	514		
		0/-45/-90/-135	50.53	514		
		0/60/-120/-180	49.47	514		
		0/-90/-0/-90	54.34	733		19,018 to 26,911
		0/-30/-0/-30	55.86	733		
		0/-45/-0/-45	55.80	733		
		0/-60/-0/-60	55.68	733		
		0/-45/-90/-135	55.82	733		
		0/-60/-120/-180	56.12	733		

In this study, the scaffold constructed exhibited a maximum porosity of 82.7 percent, compressive strength ranging from 1.76 MPa to 9.34 MPa, and structural modulus ranging from 52.2 MPa to 212 MPa after being created using a custom-defined tool path with a minimum slice thickness. Pecci et al. [37] used a random microarchitecture to create micro-bone scaffold structures. Here, the scaffold design was produced with four models, namely scaffold with pore sizes of 400 m, 500 m, 600 m, and random diameters ranging from 400 m to 600 m. The materials utilized include polylactic acid (PLA), with variations in slice thickness towards the Z-axis of 0.1 mm, 0.25 mm, and 0.4 mm, a nozzle temperature of 205 °C, and a platform temperature of 40 °C. The compressive modulus in the first section was found to be 27.8 ± 3.5 MPa and 25.3 ± 1.5 MPa for slice thicknesses of 0.25 mm and 0.4 mm, respectively.

In contrast, the compressive modulus in the second region is 1.3 ± 0.2 GPa and 1.6 ± 0.2 GPa for slice thicknesses of 0.25 mm and 0.4 mm, respectively. The scaffold has a maximum porosity of 33% for random diameters ranging from 400 m to 600 m. Sohrabian et al. [38] offered six scaffold design variants with varying geometries but the same PLA porosity. The previous study has determined that the ideal pore size and porosity values are 300 μm and around 60%, respectively. The scaffold has a pore size of 300 μm and a strut diameter of 200 μm, as well as dimensions of 10.2 × 10.2 × 15 mm and three different laydown patterns of 0/90, 0/45/135/90, and 0/60/120, as well as a shift model. The pores of the scaffold begin to break when the modulus elasticity and analytical strength of the experimental data are evaluated, and the newly established break resistance factor, the strongest shape, is found as a 0/90 pattern. The yield strength and large modulus of Young are 230.44 ± 11.2 MPa and 6289 ± 0.115 MPa, respectively. The large discrepancy between the Young' modulus 0/90 (230.44 MPa) and the 0/90 (156.56 MPa) modulus demonstrates the substantial influence of scaffold geometry on mechanical properties. Bagwan and colleagues [39] Scaffolds with varying material compositions, layer orientations, and pore sizes are built as an input parameter for previously unexplored scaffold structures employing extrusion-based additive manufacturing. In all combinations, the scaffold with the 0-90-0-90 orientation layer has Young’s modulus comparable to actual human bone. The scaffolds with 0-90-0-90 orientation layer and 350 mm pore size exhibit a higher effective Young’s modulus of roughly 30.948 GPa for a 5% HA composition.

### 4.2 Unit cell bone scaffold

Recent advancements in additive manufacturing have enabled the geometry of certain microarchitectures of cell units in cellular structures to be tailored to specific requirements. The microarchitecture of the unit cell bone scaffold is shown in Table 4. Sahmani et al. [40] created three distinct porosity scaffold models: cubic, cylindrical, and hexagonal (honeycomb) in short cylinders 20 mm in diameter and 10 mm in length. The holes should be 2–3 mm apart, with pore size variations of 1.2 mm and 0.8 mm, a nozzle temperature of 210 °C, and a nozzle diameter of 3 mm. PLA-HA composite filaments with a 30 percent HA content were employed. A tensile test with a displacement rate of 0.2 mm/min is used to measure mechanical properties (compressive stress). The compressive strength (CS) of samples having honeycomb porosities was found to be greater than that of cubic and cylindrical porosities.

**Table 4:** Microarchitecture of unit cell bone scaffold.

Author	Design	Material	Porosity (%)	PS (μm)	CS (MPa)	ME (MPa)
Sahmani et al. [40]		PLA-HA	Cub 1 = 65	Num1 = 1200	Cub 1 = 5.48	Cub 1 = 125
			Cub 2 = 70	Num 2 = 800	Cub 2 = 6.2	Cub 2 = 136
			Cyl 1 = 68		Cyl 1 = 6.52	Cyl 1 = 190
			Cyl 2 = 70		Cyl 2 = 6.7	Cyl 2 = 204
			Hex 1 = 85		Hex 1 = 7.2	Hex 1 = 350
			Hex 2 = 88		Hex 2 = 7.55	Hex 2 = 410
Cho, Gwak, and Cho [41]		PCL/nHA	C1 = 51.5 ± 1.0	C1 = 497 ± 8	NA	C1 = 62.5 ± 1.8
			C2 = 60.2 ± 1.7	C2 = 591 ± 10		C2 = 48.1 ± 4.9
			O1 = 53.3 ± 0.9	O1 = 493 ± 5		O1 = 41.3 ± 3.9
			O2 = 61.7 ± 0.9	O2 = 591 ± 12		O2 = 12.0 ± 1.1
			KD = 60.3 ± 0.8	KD = 512 ± 27		KD = 58.2 ± 7.3
Sun, Guo, and Shim [42]		PLA	G2 = 86.1 ± 1.4	G2 = 1000 ± 100	G2 = 6.1 ± 1.2	G2 = 176.7 ± 3.1
Alizadeh-Osgouei et al. [43]		PLA	G25 = 89.4 ± 1.0	G25 = 600 ± 200	G25 = 3.5 ± 0.9	G25 = 130.2 ± 2.8
			G3 = 90.3 ± 0.4	G3 = 1300 ± 200	G3 = 3.2 ± 0.5	G3 = 120.7 ± 2.5
Wojnicz, Augustyniak and Borzyszkowski [44]		ABS	NA	NA	NA	A = 117.6 ± 6.4
						B = 101.8 ± 14.2
						C = 75.6 ± 6.5
						D = 60.8 ± 9.6
						E = 59.3 ± 3.3
						F = 52.3 ± 0.7
						G = 114.2 ± 9.6
Wong et al. [45]		PEEK	Num1 = 40	Num1 = 273 ± 40	NA	NA
			Num2 = 50	Num2 = 357 ± 21		
			Num3 = 60	Num3 = 573 ± 21		
Oladapo et al. [46]		PEEK	NA	NA	NA	OT = 5790.7
						K = 165.3
						G = 291.7
						SP = 751.2

The hexagonal porosity form produces the highest compressive strength rating of 7.5 MPa. Cho, Gwak, and Cho [41] created and tested a scaffold with a two-pore kagome architectural design against four standard designs (2 have the same porosity and 2 have the same pore size). Using fused deposition modeling techniques, the scaffold has dimensions of 5 × 5 × 3.6 mm^3^, a porosity of 60%, a pore size of 500 μm, and a strand size of 1.4 mm. The compressive modulus values obtained from the tests for Conv 1, Conv 2, Offset 1, Offset 2, and dual-pore scaffolds are 62.5 ± 1.8 MPa, 48.1 ± 4.9 MPa, 41.3 ± 3.9 MPa, 12.0 ± 1.1 MPa, and 58.2 ± 7.3 MPa, respectively.

The mechanical properties of the architectural structure *kagome* two pores outperform those of other constructions with the same porosity. In terms of pore size, the compressive modulus architectural structure *kagome* two pores are the same as another scaffold. However, due to the features of the *kagome* structure, porosity is greater. Sun, Guo, and Shim [42] apply fused deposition modeling approaches to create three hybrid three-dimensional cubic lattice designs: octet structures, hybrid lattice structures 1, and hybrid lattice structures 2. According to the experimental findings, the octet structure has high stiffness and yield strength of 28.78 MPa and 1.02 MPa, respectively. This research demonstrated that by precisely regulating the topological layouts of lattice structures, it is possible to increase their energy absorption capability even further.

The gyroid scaffold’s architectural structure provides good mechanical qualities for creating bone scaffolds. Alizadeh-Osgouei et al. [43] create gyroid scaffolds (PLA) with different unit cell sizes. The porosity of the PLA scaffold varies from 86 to 90 percent, and the compressive modulus and yield strength in parallel directions are 118–180 MPa and 2–8 MPa, respectively, and 106–138 MPa and 2.5–6.0 MPa, respectively, in transverse directions. Tensile elastic modulus and yield strength are 51–63 MPa and 1.5–4.5 MPa in parallel directions, respectively, and 11–17 MPa and 1–5 MPa in transverse directions.

The greatest compressive characteristics obtained from G2 structures with large compressive yield strength and elastic modulus at 86 percent porosity are 4.6 ± 1.0 MPa and 134.8 ± 2.2 MPa in transverse directions, and 6.11 ± 2 MPa and 176 ± 73.1 MPa in parallel directions. It can be concluded that the PLA scaffold with a gyroid architectural structure has a higher compressive properties value than other gyroid constructions reported in the literature. Wojnicz, Augustyniak, and Borzyszkowski [44] used lumbar vertebrae fragments to generate 3D numerical models. The scope of this study comprised the design of nine new 3D scaffold units as well as the generation of their finite element models. Unit A is a regular cubic construction. Unit B is built on face-centered cubic construction (FCC). Unit C is formed via the body-centered cubic (BCC) topology.

In contrast, unit D is built using the truncated octahedron topology. Unit E is built using the octahedron topology. Unit F has a rhombic dodecahedron structure. The last three units were created by subtracting material from solid cubes (G, H, and I). Elements are consumed during the analytical process, and static compression tests are undertaken to determine the effective young modulus of each sample analyzed. The study’s numerical analysis revealed that 3D scaffolds used to design periodic structures, whether based on interconnected beams (units A, B, C, D, E, and F) or created by removing irregular shapes from basic solid cubes (units G, H, I), could be refined to have mechanical properties similar to trabecular bone tissue.

As a result, the scaffolds (units A, B, C, D, E, F, and H) were validated experimentally using fused deposition modeling (FMD) techniques on seven scaffolds (units A, B, C, D, E, F, and H) printed from ABS material without supporting material. Young’s experimental abs polymer modulus values were found in samples with unit H (0.19 GPa), the unit I (0.18 GPa), and unit A (0.17 GPa). To produce porous PEEK implants, Wong et al. [45] researched and designed a variety of PEEK implants with various porosities using computer-aided design/computer-aided manufacturing (CAD/CAM) and a three-dimensional (3D) printing technology fused deposition modeling.

Previous research has shown that modified porous PEEK implants have good bone compatibility, flawless stability, and good biosecurity. Furthermore, in the current study, the implants with 40%-porosity-PEEK showed the greatest potential for bone compatibility. To normalize the data, the maximum and minimum modulus of elasticity of the test results are employed. Oladapo et al. [46] investigate the design influences on bone scaffold constructs generated and developed with composite PEEK materials. Some of the microarchitectural designs developed include the octet-truss, kelvin, gyroid, and Schwarz primal. Various nanostructure approaches, such as unit cell homogenization and tensile tests, are used to study the mechanical strength of composite scaffolds and surface microstructures. The greatest and minimum moduli of elasticity of the octet-truss, kelvin, gyroid, and share primitives are 5790.7 MPa (413.9 MPa), 165.3 MPa (110.4 MPa), 291.7 MPa (260.0 MPa), and 751.2 MPa (468.5 MPa), respectively.

## 5 The FDM printing parameter

The use of fused deposition modeling (FDM) techniques for generating bone scaffolds must be accompanied by knowledge on how to select the best process parameter setting for each production. However, determining the process parameters is rather complex because of the numerous aspects that affect the operation of the 3D printer. FDM process parameters are classified as slicing, building orientation, and temperature conditions [47]. According to Sheoran and Kumar [48], printing parameters include layer thickness, build orientation, air gap, raster angle/raster orientation, extrusion temperature, print speed, infill pattern, infill density/interior infill percentage, nozzle diameter, raster width, number of contours, contour width, and contour to the contour air gap.

• Layer thickness

The amount of material deposited along the vertical axis of an FDM machine in a single pass is referred to as layer thickness or layer height. Material deposition heights are always less than the diameter of the extruder’s nozzle. The diameter of the extruder tip is fully responsible for this parameter [23].

• Build orientation

It explains how the supplied component is adapted on the build platform in terms of the three principal axes of the given machine tool (X, Y, and Z) [23]. The object’s structure usually connects build feasibility, build efficiency, and build accuracy. Anisotropic tensile strength fluctuations in FDM objects are estimated based on construction orientation. The orientation of the build has a substantial impact on the surface defects and mechanical behavior of FDM objects [49].

• Air gap

The air gap is the space (or gap) between two adjacent tool paths (or rasters) on a single layer of an FDM-made object [50]. The value of an air gap might be zero, positive, or negative. If the deposited elements are in direct touch with each other, there are no air spaces. Positive air gaps separate succeeding material deposition processes, resulting in a loosely packed structure that necessitates fast component manufacture. Because the beads partially overlap, the negative air gap produces a denser component [51].

• Raster angle/raster orientation

It is the angle (direction) of the build platform’s X-axis where extruded material is deposited. It is the angle of the raster pattern in relation to the X-axis. Typically, the raster angle ranges from 0 to 900 [48].

• Extrusion temperature

The temperature within the FDM heating nozzle before the material is extruded is referred to as the extrusion temperature [52].

• Print speed

It is the velocity at which the build nozzle crosses the XY plane while depositing material on the build platform [23].

• Infill pattern

It is the process of printing a component’s internal structure. Hexagonal, linear, and diamond patterns are examples of filling patterns [23].

• Infill density/interior infill percentage

The amount of filament printed within an item is referred to as infill density, and it has a direct impact on the print’s strength, weight, and printing time [49, 52].

• Nozzle diameter.

The diameter of the extruder’s nozzle tip is defined as the nozzle diameter [48].

• Raster width

The width of the raster is dictated by the diameter of the deposition beads. It is determined by the size of the extrusion nozzle [52].

• Number of contours

The number of contours refers to the number of solid exterior layers enclosing the internal infill pattern (or internal structure) of an FDM-produced object [48].

• Contour width

The interior structure is surrounded by the thickness of the external layers (contour layers) [48].

• Contour to a contour air gap

It refers to the air gap or space between two solid exterior layers (or contours) [48].

### 5.1 Printing process parameter on PLA

Because of its biocompatibility and bioresorbability with the human body, PLA has been widely researched for therapeutic applications [53]. Ariffin et al. [54] study the ability of an open-source 3D printer to produce bioresorbable scaffolds with fully connected channel networks. They adapted the nozzle of a well-known open-source 3D printer to print PLA and PMMA materials. According to the findings, the ideal temperature for extruding PLA material is 190 °C, whereas the optimal temperature for extruding PMMA material is 200 °C.

Carlier et al. [55], on the other hand, study the feasibility of manufacturing implanted devices using 3D printing Fused deposition modeling (FDM) technology. The impact of deposition temperature, deposition rate, and layer thickness on the printing process and device physical properties were studied. The filaments were made of pure polylactic acid (PLA) and plasticizer blends. According to the results, using a high layer thickness, a low temperature, and an acetyl triethyl citrate plasticizer boosted ductility. The temperature was raised, the layer thickness was reduced, and triacetin was added to increase adhesion.

Similarly, Khosravani and Reinicke et al. [56] investigated two printing parameters in 3D-printed part production: (a) raster layup and (b) printing speed. A number of studies were conducted to demonstrate the impact of the printing parameters on stiffness and strength. In the fused deposition modeling (FDM) method, polylactic acid (PLA) material was employed to generate specimens. The collected data revealed that raster angle has an effect on stiffness and strength. The highest and lowest strengths were found for raster angles of 0 and 90, respectively. Lyu et al. [57] investigated the effect of 3D printing parameters such as layer thickness, nozzle temperature, printing speed, and platform temperature using an orthogonal experimental design. The results showed that when the layer thickness was 0.15 mm, the printing speed was 50 mm/s, the nozzle temperature was 200 °C., and the platform temperature was 50 °C., the 3D printing specimen operated optimally.

Deomore and Raykar [58] use the VIKOR approach to optimize FDM process parameters using PLA material. Three essential FDM process parameters are chosen for optimization: layer thickness, infill percentage, and speed. The optimal process parameters, according to the results, are a layer thickness of 0.3 mm, a speed of 100 mm/h, and an infill of 55%. Samykano [59] investigates the tensile behavior of PLA using FDM 3D printing and proposes a mathematical model for predicting its properties. 1.75-mm PLA filament was used to create this specimen. According to the results, infill percent has the greatest influence on ultimate tensile strength, followed by raster angle and layer thickness. However, infill percentage and layer thickness have a bigger impact on fracture strain, elastic modulus, and toughness. Hikmat, Rostam, and Ahmed [60] studied the effect of various printing parameters on tensile strength using polylactic acid (PLA) filament, including build orientation, raster orientation, nozzle diameter, extruder temperature, infill density, shell number, and extruding speed. The results showed that the selected process factors had a significant effect on component strength, with only three of them statistically significant: build orientation (on-edge), nozzle diameter (0.5), and infill density (100 percent).

### 5.2 Printing process parameter on PCL

Fard et al. [61] optimize the FDM 3D printing processing settings for nanocomposites (PCL/nHA/CNW). For FDM operations, four level-three parameters were established to identify the ideal print speed, nozzle temperature, build plate temperature, and fan speed. The results show that the best parameters are build plate temperature (30 °C), extruder temperature (100 °C), fan speed (100%), and extruding speed (15 mm/s). Ariadna et al. [62] investigated the optimization of the open-source, low-cost 3D extrusion machine RepRap, which was used to create PCL scaffolds suitable for 3D cell culture. Several process factors in the fabrication of scaffolds and cell cultures were investigated to confirm the findings. The results show that the optimal parameters are 0.35 mm nozzle tip size, 0.3 mm layer height, 10 mm/s extrusion speed, and 85 °C extrusion temperature. Table 5 depicts the printing parameters of PLA and PCL materials.

**Table 5:** Printing parameter of PLA and PCL material.

Author	Material	Printing Parameter	Mechanical Properties
Ariffin et al. [54]	PLA	Printing temperature = 190 °C	Compressive strength
		Bead temperatures = 50 °C	Porosities
		Printing speed = 60 mm/s	
Carlier et al. [55]	PLA	Printing temperature = 190 °C	Tensile strength
		Layer thickness = 0.1 mm	Elongation at break Elastic modulus
		Deposition rate = 88 mm/s	
Khosravani and Reinicke et al. [56]	PLA	Nozzle temperature = 215 °C	Tensile strength
		Layer thickness = 0.4 mm	Stiffnes
		Bed temperature = 55 °C	Elastic modulus
		Printing speeds = 20 mm/s	
		Raster orientations = 0°	
Lyu et al. [57]	PLA	Layer thickness = 0.15 mm	Yield strength
		Printing speed = 50 mm/s	Elongation at break
		Nozzle temperature = 200 °C	Dimensional accuracy
		Platform temperature = 50 °C	
Deomore and Raykar [58]	PLA	Layer thickness = 0.3 mm	Time
		Speed = 100 mm/h	Weight of Product
		In fill percentage = 55%	Filament length
Samykano [59]	PLA	Layer height = 0.3 mm	Ultimate tensile strength
		Raster angle = 40 °C	Fracture strain
		Infill density = 80%	Elastic modulus
			Yield strength
			Toughness
Hikmat, Rostam, and Ahmed [60]	PLA	Build orientation = on-edge	Tensile strength
		Raster orientation = 30/-60°	
		Nozzle diameter = 0.5 mm	
		Extruder temperature = 220 °C	
		Infill density = 100%	
		Extruding speed = 20 mm/s	
Fard et al. [61]	PCL	Nozzle temperature = 100 °C	Structural integrity
		Print speed = 15 mm/s	Compressive strength
		Build plate temperature = 30 °C	
		Fan speed = 100%	
Ariadna et al. [62]	PCL	Nozzle tip size = 0.45 mm	Visual screening
		Layer height = 0.3 mm	
		Extruding speed = 10 mm/s	
		Extrusion temperature = 85 °C	

## 6 The FDM material bone scaffold

Scaffold materials must be carefully selected to meet the criteria for effective clinical translation. They must be biocompatible and bioactive and have adequate mechanical strength in vivo. FDM technology uses a limited number of biocompatible and bioactive materials. Most FDM filament materials are not environmentally friendly because they are petroleum-based and may emit toxic materials during the printing process, which has a negative impact on health and the environment [63]. Developing a biobased filament for FDM is gaining popularity because it not only reduces the use of petroleum-derived plastic but also lowers filament costs [64]. Many studies have been conducted on the use of synthetic polymers derived from renewable resources such as corn, corn molasses, and beet sugar levels as biomaterials in FDM technology. Synthetic polymers are advantageous because they allow the user to modify mechanical and biological properties as well as control the degradation rate by varying the functional groups linked to the main polymer chain and the number of monomers employed in the polymer’s synthesis. Some of the most commonly used synthetic polymers are polyglycolic acid (PGA), polylactic acid (PLA), polylactic glycolic acid (PLGA), and polycaprolactone (PCL) [65].

Furthermore, these polymers do not provoke an immunological response or trigger immune rejection. Another advantage is that these polymers are simple to produce and have high mechanical characteristics that can withstand tissue collapse. As a result, they’ve been used in bone tissue engineering as scaffolds [66]. Combining two or more materials with different compositions and qualities can produce composites with tunable physical and chemical properties, as well as improved mechanical and bioactivity. Polymer-based composites have grown in popularity in recent years due to their increased durability and wide range of processing methods. As promising bone regeneration scaffolds, various biodegradable polymers have been found [67].

### 6.1 Polylactic acid (PLA) biocomposite

PLA is a biodegradable, biocompatible, and compostable polyester derived from renewable resources such as corn, corn molasses, and beet sugar levels [68]. Thermal stability, cytocompatibility, and non-toxic degradation products distinguish PLA. It is available in several forms, including poly-l-lactide acid (PLLA) and poly-d-lactide acid (PDLA), the L/D ratios of which can be adjusted to optimize the degradation rate of the materials [8]. This polymer is classified as a thermoplastic aliphatic polyester and serves as the primary organic raw material in additive manufacturing using FDM technology [49]. Despite these excellent characteristics, PLA is a brittle polymer with low toughness, one of the primary limitations to its sustained development [53].

Furthermore, PLA’s low biodegradability and hydrophobicity continue to limit its usage in the biomedical area [69]. Several research groups have proposed several ways, including copolymerization, polymer blending, and polymer compositing, to overcome the drawbacks of PLA and generate improved materials for various applications [70]. Ferri et al. [71] created composite materials using a PLA matrix and HA as an osteoconductive filler. They obtained composites with varying HA contents ranging from 10 to 30% by weight. The maximum Young’s modulus of PLA-HA composites containing 20–30 wt% HA is 28% greater than that of pure PLA, showing an increase in stiffness.

As a result, Wu et al. [72] printed the trabecular models with PLA/HA composites and examined the morphological and mechanical properties of the printed models. Filaments with mineral concentrations of 5%, 10%, and 15% HA were generated. The addition of HA to PLA raised the elastic modulus but had no effect on the compressive strength. Diez-Escudero et al. [73] studied the physicochemical and biological features of polylactic acid (PLA) scaffolds in conjunction with hydroxyapatite (HA). HA was either added to the polymer matrix or employed as a coating, resulting in 15% and 2% wt., respectively. The results reveal that triangular and hexagonal pores increased mineralization, but only when HA was present, either as a coating or as a composite with PLA.

Bernardo et al. [74], on the other hand, increased the HA loading (20–30%) in PLA composite filaments to improve the bioactivity of 3D printed bone tissue engineering scaffolds. According to the findings, HA lowered the water contact angle, boosting the hydrophilicity of the scaffolds. Wang et al. [75] used 3D printing to create porous bone tissue scaffolds with n-HA gradients of 0%, 10%, 20%, 30%, 40%, and 50%. The mechanical properties of these specimens were evaluated. The printed models from the Pn0 to Pn30 groups retained structural integrity after the pressure test, demonstrating their elasticity. The printed specimens became brittle and lost shape when the n-HA ratio exceeded 40%. The amount of n-HA in the PLA/n-HA is based on the porosity test results. The printed specimens became brittle and lost shape during compression when the n-HA ratio approached 40%. The amount of n-HA in the PLA/n-HA wet composites had no effect on porosity, according to the porosity test results.

**Figure 5:**
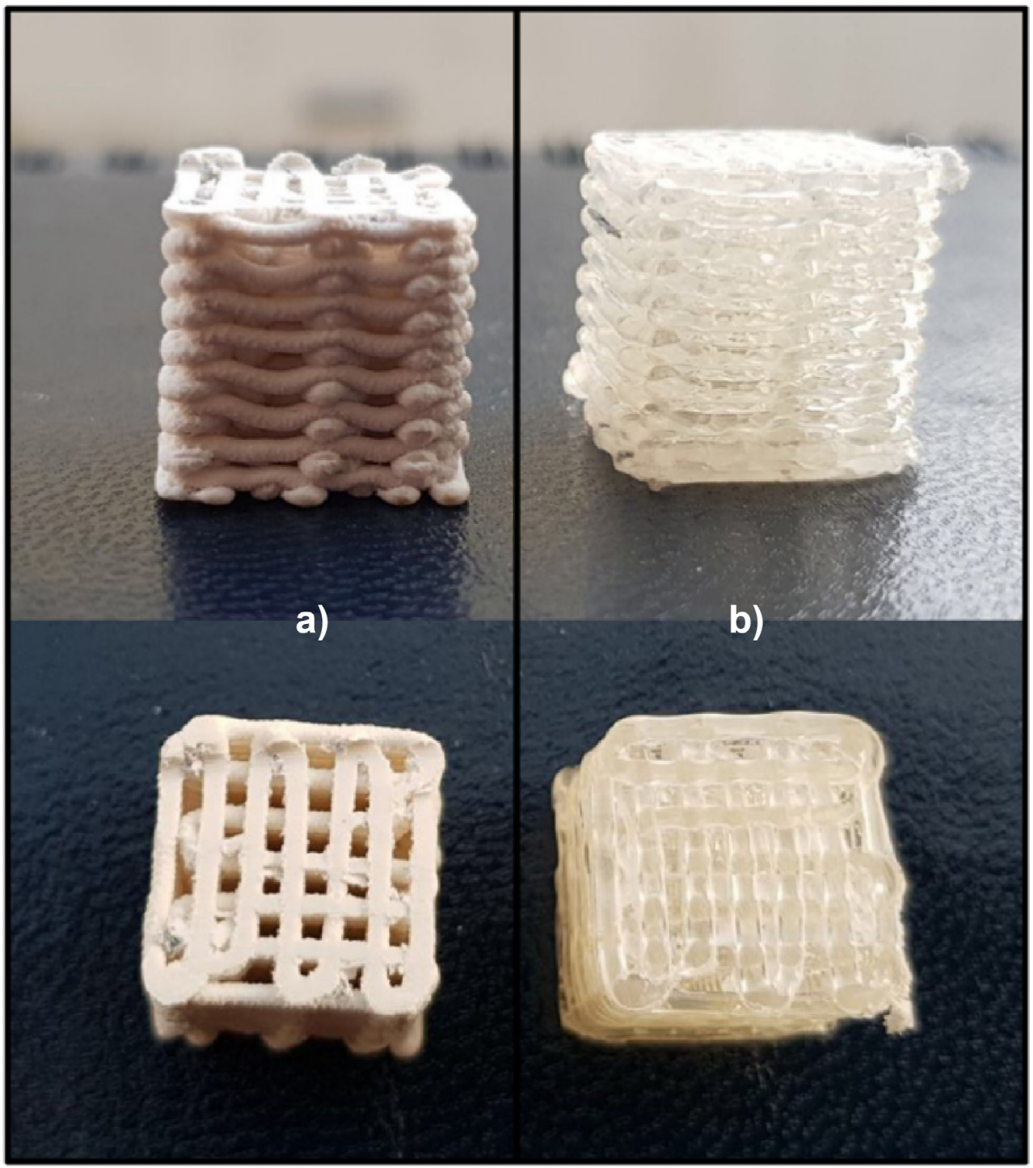
3D print sample: (a) PLA/sdHA and (b) PLA porous [76].

Similarly, Corcione, Gervaso, and Scalera [76] developed 3D printed scaffolds for bone tissue engineering employing high-loaded filaments (50 % wt. HA) at the composite’s PLA matrix. An FDM printer was then used to manufacture three-dimensional samples with a theoretical porosity of 50%. Furthermore, a little bending of the struts deposited by the printer was discernible with the naked eye in both PLA and PLA/sdHA samples, however this deflection was significantly more pronounced in PLA/sdHA than in PLA as shown in Figure 5(a) and (b). The rigidity of PLA and PLA/sdHA scaffolds was 238.98 19.05 MPa and 124.04 25.21 MPa, respectively. The higher the porosity of composite specimens, the lower the stiffness under compression.

### 6.2 Polycaprolactone (PCL) biocomposite

PCL is an aliphatic semi-crystalline polymer having a glass transition temperature of −60 °C and a melting temperature ranging from 59 to 64 °C. Bulk PCL has a tensile strength of about 25–43 MPa and an elastic modulus of about 330–360 MPa. Blending a PCL scaffold with various ceramic materials improves its mechanical characteristics for bone tissue engineering [77]. PCL is a polymer that can be employed in a variety of biomedical research applications. PCL or PCL/hydroxyapatite scaffolds, for example, have been created using the exact extrusion deposition approach. The mechanical characteristics, pore size, and interconnectivity of these materials make them excellent for bone tissue manufacturing. They have the necessary mechanical properties, as well as the appropriate pore size and interconnectivity [78].

Xu et al. [79], on the other hand, investigated 3D artificial bones that mimic real goat femurs utilizing PCL and PCL/HA composite material. The results reveal that 3D PCL/HA artificial bones outperform PCL in terms of cell biocompatibility, biodegradation, and new bone formation capacity. Kim et al. [80] create and characterize biocompatible PCL/HA filaments for bone scaffolds using FDM 3D printing. Some filaments with varying quantities of HA, ranging from 5% to 10%, 15%–20%, and 25%–25%, were created. Tensile testing shows that as the HA content of the composite filament increases, so does the fracture strain and tensile strength. Pierantozzi et al. [81] investigated how the design and formulation of PCL, HA, and SRA composites affect mechanical and biological properties. The results showed that the ceramic phase (first-seating of 20%) in the polymer matrix had little effect on the Scaffold PLC Young modulus values.

**Figure 6:**
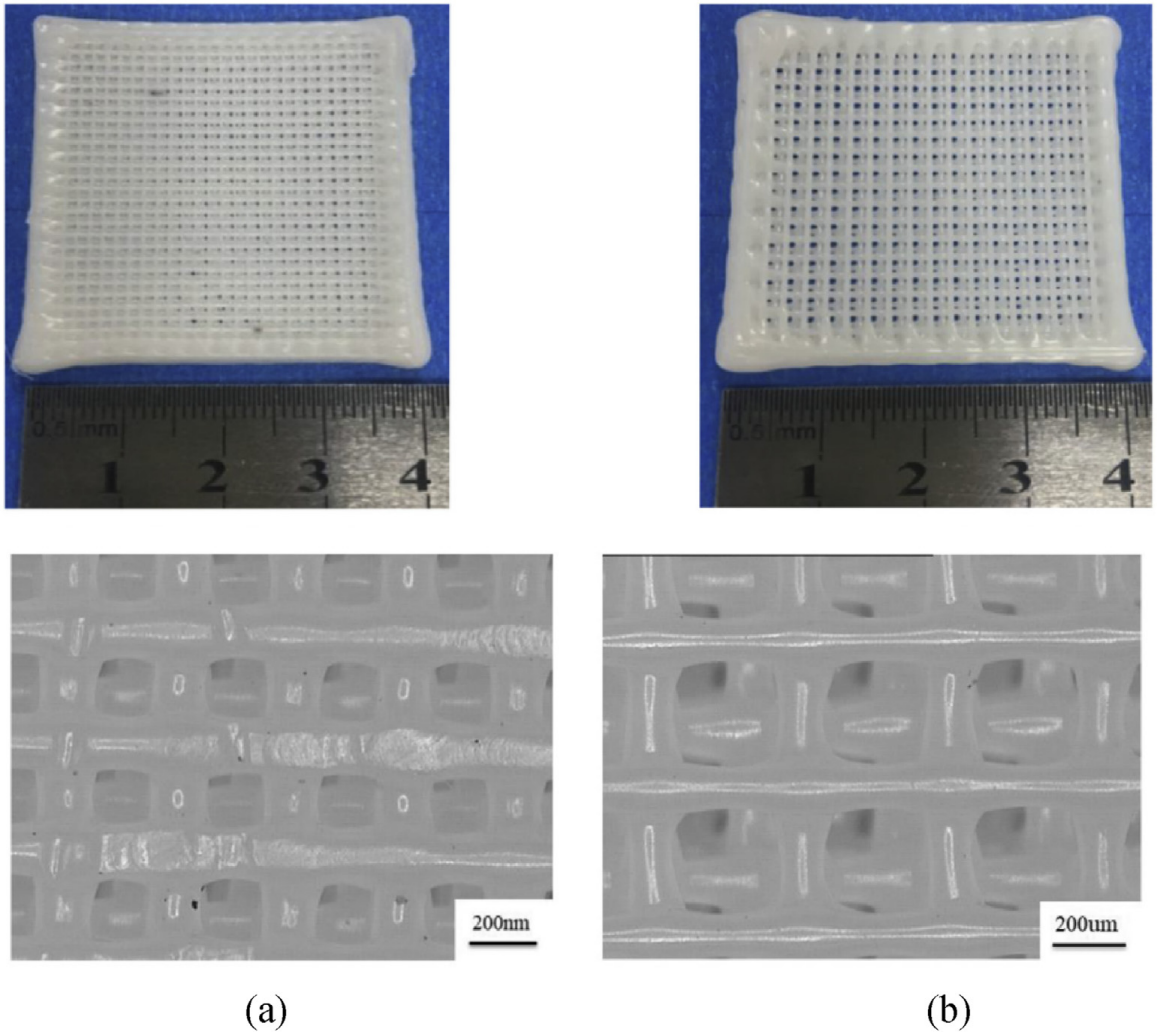
The composite bone scaffold: (a) micro-HA/PCL, (b) nano-HA/PCL [82].

Jiao et al. [82] investigate the internal structure and mechanical properties of a hydroxyapatite/polycaprolactone scaffold. In this work, nano-ha/PCL and micro-ha/PCL with 20% ha were used as raw materials. The nano-HA/PCL and micro-HA/PCL composite bone scaffolds were capable of forming a predetermined pore configuration with interconnected pores as shown in Figure 6(a) and (b). The results reveal that HA particles in Scaffold Nano-Ha/PCL scaffolding may be dispersed evenly, however, HA particles in Scaffold Micro-Ha/PCL scaffolding are lumpy. Nano-Ha/PCL scaffolds have greater tensile and flexural strength than micro-ha/PCL scaffolds. Fard et al. [61] design, develop, produce, and characterize PCL/nHA/CNW nanocomposite filaments for bone scaffold manufacturing using FDM technology. New nanocomposites are being tested for mechanical, biological, and biodegradability properties. The results demonstrated that CNW filaments somewhat improved the mechanical qualities of 3D-printed objects, while a nanocomposite with 3% CNW content had a substantial effect on cell proliferation and scaffold attachment properties.

Momeni et al. [83] investigated the mechanical and microstructural properties of PCL-based composites with fluorapatite (nFA) nanoparticles. Mechanical testing revealed that adding up to 20% nFA to PCL increased tensile and yield strength while decreasing elongation at yield and failure points and increasing Young modulus. The mechanical properties of the PCL/20nFA composite were the best. Tensile strength and young modulus of the material were increased by 30% and 179 %, respectively. Meanwhile, PCL/20nFA elongation was reduced by 70% when compared to naked PCL. In terms of mechanical properties, PCL/20nFA may be considered a suitable composite for bone tissue regeneration based on the data obtained. Table 6 depicts the composition and parameters of biomaterials-based FDM bone scaffolds.

**Table 6:** Composition and parameters of biomaterials-based 3D printed bone scaffolds.

Author	Material	Compositions	Parameters
Ferri et al. [71]	PLA	PLA	Hardness
		PLA-10HA	Tensile strength
		PLA-20HA	Tensile modulus
		PLA-30HA	Elongation at break
			Flexural strength
			Flexural strength
Wu et al. [72]	PLA	PLA and 5 wt% HA	Compressive strength
		PLA and 10 wt% HA	Elastic modulus
		PLA and 15 wt% HA	Pullout load
Diez-Escudero et al. [73]	PLA	PLA	Thermal characterization
		PLAcHA	Physicochemical characterization
		PLA15H	Biological characterization
Bernardo et al. [74]	PLA	PLA	Physical-chemical characterization
		80%PLA: 20%HA	Thermal analysis
		75%PLA: 25%HA	Wettability
		70%PLA: 30%HA	
Wang et al. [75]	PLA	PLA	Characterization of PLA/n-HA
		10% n-HA/PLA	In vitro experiment
		20% n-HA/PLA	In vivo experiment
		30% n-HA/PLA	
		40% n-HA/PLA	
		50% n-HA/PLA	
Corcione, Gervaso, and Scalera [76]	PLA	PLA	Compressive strength
		PLA/sdHA	Stiffnes
			Morphology
			Porosity
			Glass transition temperature.
			Melting temperature
Xu et al. [79]	PCL	PCL	Porosity
		70%PCL:30%HA	Compressive Strength
			Elastic Modulus
			In vitro experiment
Kim et al. [80]	PCL	PCL/HA 5 wt.%	Morphology
		PCL/HA 10 wt.%	Tensile Strength
		PCL/HA 15 wt.%	Fracture Strain
		PCL/HA 20 wt.%	
		PCL/HA 25 wt.%	
Pierantozzi et al. [81]	PCL	100% PCL	Porosity
		90% PCL:10% HA	Compressive stress
		90% PCL: 10% SrHA	Elastic Modulus
		80% PCL: 20% HA	In vitro experiment
		80% PCL: 20% SrHA	
Jiao et al. [82]	PCL	PCL	Microstructures
		20% micro-HA/PCL	Crystallization temperature
		20% nano-HA/PCL	Micropore structure
			Porosity
			Tensile strength
			Flexural strength
Fard et al. [61]	PCL	PCL	Compressive strength
		P-nHA1%	Apparent modulus
		P-nHA2%	TGA results
		P-nHA3%	FTIR results
		P-CNW1%	Cell viability results
		P-CNW2%	Biodegradation rate
		P-CNW3%	
		P-nHA1.5%-CNW1.5%	
		P-nHA1%-CNW2%	
		P-nHA2%-CNW%	
Momeni et.al [83]	PCL	PCL	XRD result
		PCL/10nFA	FTIR result
		PCL/20nFA	SEM image
		PCL/30nFA	Yield strength
			Young modulus
			Tensile strength
			Elongation

## 7 Conclusion and research gap

The bone scaffold can be built swiftly and according to the materials chosen using additive manufacturing or 3D printing technology. Because of its low cost, small size, capacity to construct complicated structures, and lack of organic solvents, FDM is one of the most commonly used 3D printing technologies in biomaterial research. When fabricating bone scaffolds with the FDM method, three factors must be considered: the design architecture, the materials, and the process parameters. Microarchitecture design is an important step in the production of bone scaffolds. Bone scaffold architecture is classified into two categories. The first is based on unit cell designs, whereas the second is based on entire designs. Architectural designs such as Kagome lattice, octet structures lattice, and gyroid lattice can have compressive properties as excellent as the bone scaffold.

3D printers enable the bone scaffold to be built swiftly and in accordance with the various materials chosen. Polycaprolactone (PCL) composite and polylactic acid (PLA) composite are the most commonly used materials in the development of bone scaffolds. Other materials that can be researched further include poly (3-hydroxybutyrate-co-3-hydroxyvalrate) (PHBV) and PEEK. The material composition employed in the manufacture of bone scaffolds can have an impact on mechanical and biological qualities. The addition of hydroxyapatite (HA) to polycaprolactone (PCL) can improve mechanical and biological characteristics. The inclusion of hydroxyapatite (HA) composition in polylactic acid (PLA) can improve biological qualities while decreasing mechanical properties. However, several studies have indicated that increasing the percentage of the composite can improve mechanical properties.

Several process parameters have a major impact on the features of the construction part and its production efficiency. Layer thickness, raster angle, build orientation, infill density, printing speed, infill pattern, extrusion temperature, raster width, nozzle diameter, contour width, contour to contour air gap, contour numbers, and air gap are the critical process parameters.

Some conclusions and research gaps of all articles analyzed are as follows:

• Understanding of scaffold microarchitecture design with the best compressive characteristics is required. Few studies of scaffold microarchitecture design have been conducted to compare mechanical and biological qualities in the same porosity and composite materials in order to determine the most powerful architectural designs.
• It is envisaged that determining the optimum material composition will result in material compositions with mechanical and biological qualities similar to those of actual bones. Polycaprolactone (PCL) and polylactic acid (PLA) are materials with bone replacement needs, however research on polycaprolactone alloys (PCL), polylactic acid (PLA), and hydroxyapatite (HA) is still scarce.
• 3D-Printing parameters have a large influence on mechanical qualities. They must be tuned, although studies on parameter printing have largely focused on tensile parameters, with compressive parameters being uncommon as critical factors on bone scaffolds.
• The mechanical and biological qualities of the bone scaffold will be affected by the use of different thermoplastic materials in FDM procedures. Optimization of printing settings, which is commonly done with Polylactic Acid (PLA) materials, is also infrequently done with other composite materials such as hydroxyapatite (HA) and polycaprolactone alloys (PCL).

## Declarations

### Author contribution statement

All authors listed have significantly contributed to the development and the writing of this article.

### Funding statement

A.P Bayuseno was supported by Deputy for Strengthening 10.13039/100006190Research and Development, the Ministry of Research and Technology, Higher Education, the Republic of Indonesia for research funding under Ph.D [Number: ∗∗∗/UN7.P4.3/PDD/2021].

### Data availability statement

No data was used for the research described in the article.

### Declaration of interest’s statement

The authors declare no conflict of interest.

### Additional information

No additional information is available for this paper.
